# Standard Deviational Ellipse (SDE) models for malaria surveillance, case study: Sukabumi district-Indonesia, in 2012

**DOI:** 10.1186/1475-2875-11-S1-P130

**Published:** 2012-10-15

**Authors:** Tris Eryando, Dewi Susanna, Dian Pratiwi, Fajar Nugraha

**Affiliations:** 1 Department of Biostatistics and Population Studies, Universitas Indonesia, Kampus Ul Depok, 16424, Indonesia; 2 Department of Environmental Health, Universitas Indonesia, Kampus Ul Depok, 16424, Indonesia; 3 Center For Biostatistics and Health Informatics Studies, Universitas Indonesia, Kampus Ul Depok, 16424, Indonesia; 4 Center For Biostatistics and Health Informatics Studies, Faculty of Public Health, Universitas Indonesia, Kampus Ul Depok, 16424, Indonesia

## Background

Sukabumi District has been a malaria endemic area for 8 years. In 2004 an outbreak of malaria occurred, and more than 250 positive malaria cases are reported every year. Malaria surveillance data is still in the form of tabular data [1], therefore it is necessary to find models to support the malaria surveillance, based on spatial mapping and analysis with the use of Standard Deviational Ellipse (SDE) models. Malaria distribution maps are a strategy to target resource distribution and to focus the control program [2].

## Materials and methods

The research used a cross-sectional study. Data collection through Global Positioning System (GPS) plotting, surveys and interviews based on positive malaria cases 2011-2012 derived from public health centers. Analysis of data using overlay analysis with physical environment variables, spatial statistical analysis and SDE.

## Results

The highest malaria incidence occurred at ambient temperature 22-25 degrees Celsius (70%), with an altitude of 500-1000m (71%) in the south hills and mountainous areas; rainfall is 3453-3846mm (50%) in the northern areas, the distance from breeding place less than 500m (84%), and interaction the physical environment with vector enabling an outbreak risk. Mean center of gravity was the center of the distribution of cases was Longitude: 106.602721 and Latitude: -7.118190. The locations of respondents were quite close together; this could mean that malaria was spread evenly due to import cases. The rotation angle of SDE was 58.524624 degrees clockwise and the area of ellipse was 146,109,759 square meters. Standard deviational ellipse as an overview of the standard deviation of the distribution showed that the length of the X axis was 15,936.83 m and the Y axis was 11,673.13 m, the ratio between the X and the Y axis wass equal to 1.3653 (Table 1). Direction of the axis of standard deviation ellipses appeared that the skewed distribution towards the northwest-southeast. Rainfall and temperature anomalies were two of the major environmental factors triggering epidemics in warm semi-arid and high altitude areas [3-5]. Physical environment of Sukabumi District supported the development and metabolism of vectors. The map provided an initial description of the geographic variation of malaria, and might assist in formulating various methods of intervention [6].

**Table 1:** Mean Center and SDE

Variable	Sub Variable	X	Y
Mean Center	Minimum	106.487241	-7.232089
	Maximum	106.7401	-7.018312
	Mean	106.602721	-7.11819
	Standard Deviation	0.041497	0.047272
	Geometric Mean	106.602713	-7.118034
	Harmonic Mean	106.602705	-7.117879
SDE	SD along new axis	7968.41m	5836.57m
	axis length	15936.83m	11673.13m

## Conclusion

Standard Deviational Ellipse (SDE) models can be used to gain a better understanding of the geographical aspects of the phenomenon and identify the cause of an event, based on specific geographic patterns.
